# A comparative study of shoulder replacement outcomes using linked national registry and hospital data from England and Denmark

**DOI:** 10.1186/s12916-025-04003-3

**Published:** 2025-03-26

**Authors:** Epaminondas Markos Valsamis, Josefine Beck Larsen, Theis M. Thillemann, Stephen E. Gwilym, Gary S. Collins, Inger Mechlenburg, Jonathan L. Rees

**Affiliations:** 1 Botnar Research Centre, Nuffield Department of Orthopaedics, Rheumatology and Musculoskeletal Sciences, , University of Oxford, Oxford, OX3 7LD UK; 2 Department of Clinical Medicine, Aarhus University, Palle Juul-Jensens Boulevard 99, Aarhus N, 8200 Denmark; 3 Department of Orthopaedic Surgery, Aarhus University Hospital, Palle Juul-Jensens Boulevard 99, Aarhus N, 8200 Denmark; 4 Centre for Statistics in Medicine, Nuffield Department of Orthopaedics, Rheumatology and Musculoskeletal Sciences, University of Oxford, Oxford, OX3 7LD UK; 5 Research Center for Prevention and Health Promotion, VIA University College, Hedeager 2, Aarhus N, 8200 Denmark; 6 NIHR Oxford Biomedical Research Centre, Oxford, UK

**Keywords:** Shoulder replacement, Patient outcomes, Routinely collected healthcare data

## Abstract

**Background:**

The incidence of shoulder replacement surgery continues to rise internationally. The aim of this study was to compare revision surgery, reoperations and serious adverse events after shoulder replacement surgery in England and Denmark.

**Methods:**

Linked National Joint Registry and NHS Hospital Episode Statistics of England, and linked Danish Shoulder Arthroplasty Registry and Danish National Patient Registry data were available from 1 April 2012 to 31 December 2020. All primary shoulder replacements in adult patients were included. Revision surgery, reoperations and serious adverse events were compared between the two countries, and stratified by procedure type and surgical indication. The risk of revision and serious adverse events were adjusted for age, sex and comorbidities, using flexible parametric survival models and logistic regression models, respectively.

**Results:**

A total of 41,471 and 9,268 primary shoulder replacement procedures were analysed from England and Denmark, respectively. The mean patient age in Denmark was 70.6 years (SD 10.1) and in England 72.6 years (SD 9.9). Danish patients had a lower risk of serious adverse events (4.5%) compared to patients in England (5.6%), but a slightly higher risk of re-operations by 1 year (Denmark 2.3% [95% CI 2.0% to 2.6%], England 1.7% [95% CI 1.6% to 1.8%]). There was a slightly lower risk of revision joint replacement surgery by 8 years in Denmark (5.1% [95% CI 4.5% to 5.8%]) compared to England (5.7% [95% CI 5.4% to 6.1%]). The reverse total shoulder replacement had a higher revision rate in Denmark, but the anatomical total shoulder replacement and humeral hemiarthroplasty had lower revision rates. Denmark had a considerably higher revision rate for patients having surgery for acute trauma. These results remained the same after adjusting for age, sex, and the Charlson Comorbidity Index.

**Conclusions:**

While there was variation in the demographics of patients having shoulder replacement surgery in England and Denmark, differences in serious adverse events and revision rates were observed despite case-mix adjustment. Some of this variation might be attributed to the differences seen in the use of different procedures for different surgical indications between the two countries.

**Supplementary Information:**

The online version contains supplementary material available at 10.1186/s12916-025-04003-3.

## Background

The incidence of shoulder replacement surgery is increasing rapidly around the world. Despite this surge, there has been variation in the use of different shoulder replacement procedures and in the indications for surgery across countries [1]. This disparity can be partly attributed to differences in healthcare systems, surgeon preferences and patient demographics. Variation in practice is also attributable to the absence of high-quality evidence to guide surgeons in their choice of shoulder replacement procedures according to the type of joint disease and patient factors identifiable pre-operatively [2, 3]. The desire for comparative effectiveness of the different types of shoulder replacement procedures has been identified as one of the top ten research priorities by patients, carers and clinicians as stakeholders look to make informed choices in healthcare [4].

While reporting variation in incidence and surgical practice within a country is crucial for quality control, much greater value lies in comparing patient outcomes across different countries and healthcare systems. Comparisons between countries can highlight the effects of different healthcare systems, and the influence of evidence on local surgeons. It can also identify system differences that may preclude future interventional studies between the two countries. Outcome-based comparisons can provide valuable insights to support policy planning, decision-making, and ultimately, the improvement of patient care globally [5, 6]. Data storage and sharing restrictions have posed significant challenges in conducting international comparison studies [7]. In Orthopaedics, the only combined published studies come from the Nordic Arthroplasty Register Association (NARA) that unites joint registries from Denmark, Sweden, Finland and Norway. NARA have published comparative studies for hip and knee replacements, focusing on the outcome of revision surgery [8, 9]. However, no cross-country study has yet been undertaken in any field of Orthopaedics using linked joint registry and hospital data to allow for the comparison of other important outcomes to patients such as medical complications after surgery.

The aim of this study was to use linked joint registry and hospital data from England and Denmark to assess whether revision surgery, reoperations and serious adverse events after shoulder replacement surgery were the same or different between England and Denmark.

## Methods

### Data sources

The English dataset comprised linked data from the National Joint Registry (NJR), NHS Hospital Episode Statistics (HES) Admitted Patient Care, and Civil Registration Mortality data [10, 11]. Data submission to the NJR is mandatory for all private and public hospitals and includes details on patients, surgeons, and operations. The HES database records all inpatient and day case activity in England and contains a range of demographic data, medical diagnoses, procedural information, and administrative data. HES data are used to ensure accurate reimbursement to NHS providers for their activities. The Danish dataset comprised linked data from the Danish Shoulder Arthroplasty Registry (DSR), the Civil Registration System (CRS) and the Danish National Patient Registry (DNPR) [12–14]. All public and private hospitals are required to submit data to the DSR which contains information on surgical procedures. The CRS contains information on mortality and migration. The DNPR is used by healthcare authorities to reimburse Danish hospitals for their activity and records medical diagnoses, comorbidities, and administrative data for any inpatient or outpatient activity, and was revised in 2019.

### Selection criteria

Linked data from England and from Denmark from 1 April 2012 to 31 December 2020 were available for this study. All primary shoulder replacements in patients aged 18 to 100 years were included. Duplicates or invalid records (i.e., inconsistent surgical history) were excluded. Surgeries for malignancy were excluded. The unit of analysis was the procedure rather than the patient, so a patient who had undergone both a left and right-sided shoulder replacement would appear as two separate procedures. All selection criteria and definitions, including the relevant codes for patient outcomes, were agreed a priori.

### Outcomes

The primary outcome was revision surgery, defined as a procedure that involves adding, removing, or modifying one or more components of a joint prosthesis [15]. For the English data, NJR records were used to identify revisions, whereas for the Danish data, DSR records were used to identify revisions.

Secondary outcomes were identified from HES for England and from the DNPR for Denmark. These included reoperation within 12 months and serious adverse events (SAE) within 90 days of primary surgery. Some patients undergo a different type of shoulder procedure on the same side which does not involve changing the shoulder replacement implants. These procedures are not considered revisions, but they are reoperations and are an important outcome for patients. To capture all subsequent surgery that is relevant to the primary shoulder replacement, any of the following reoperations occurring on the same shoulder and on a separate occasion to any revision surgery, were recorded: subacromial decompression or acromioclavicular joint excision, or both; rotator cuff repair; manipulation under anaesthesia or release, or both; washout or debridement; synovectomy; osteomyelitis surgery; complex reconstruction; bone resection; arthroscopy or surgery for instability; reduction of dislocation; or fixation of a periprosthetic fracture. Office of Population Censuses and Surveys classification of surgical operations and procedures, fourth revision (OPCS-4) and the Nordic Medico-Statistical Committee (NOMESCO) procedure codes were used to identify reoperations in England and Denmark, respectively. SAEs were defined as medical complications severe enough to require admission to hospital, identified using ICD-10 (International Classification of Diseases, 10th revision) codes: pulmonary embolism, myocardial infarction, lower respiratory tract infection, acute kidney injury, urinary tract infection, cerebrovascular events, and all-cause death [16]. When reporting and analysing SAEs, procedures after 2 October 2020 were excluded to ensure all patients had 90 days of follow-up. See Additional file 1 for lists of procedure and diagnosis codes used.

Patients were censored after death (and after emigration in the Danish data), and data for revision and reoperation were reformatted into time-to-event variables suitable for survival analysis. The estimand was the net failure of the implant, so the Kaplan–Meier estimator was used [17, 18]. SAEs were treated as binary variables representing the presence of the event.

### Statistical analysis

Baseline patient demographics were reported for each country. Analyses of primary and secondary outcomes were stratified by grouped surgical indication and by procedure type. The following groups of surgical indications were generated: elective osteoarthritis; cuff tear arthropathy (also including rotator cuff tear and inflammatory arthropathy); acute trauma; other (including trauma sequelae and avascular necrosis). The following three shoulder replacement procedure types were all included: humeral hemiarthroplasty (HA); anatomical total shoulder replacement (TSR); and reverse total shoulder replacement (RTSR).

Unadjusted Kaplan–Meier survival curves were generated for revision and reoperation, and proportions were reported for 90-day SAE. Due to data restrictions imposed by the Danish authorities, smoothed Kaplan–Meier plots were reported. Models were then generated for revision and 90-day SAE within each country’s dataset, adjusting for clinically important covariates. Flexible parametric survival models were used for revision, adjusting for age, sex, categorised Charlson Comorbidity Index (CCI), and either procedure type or grouped surgical indication. Procedure type and grouped surgical indication were additionally adjusted for as a time-varying covariate for models used to predict survival probabilities by procedure type and grouped surgical indication, respectively. The degrees of freedom for the baseline hazard function and time-varying covariates for each model were chosen based on graphical inspection and the Akaike information criterion (AIC) and Bayesian information criterion (BIC). Age was modelled using restricted cubic splines with three knots based on graphical inspection, the AIC and BIC. Logistic regression models were used for 90-day SAE, adjusting for the same confounding variables.

There were no missing data in any covariate or outcome in the English dataset, but procedure type was missing in 21 procedures (2.3%) in the Danish dataset, so a complete case analysis was undertaken. Stata (V.18) was used for all analyses [19].

## Results

### Patient characteristics

The English and Danish datasets comprised 41,471 and 9,268 procedures, respectively (Additional file 1). Compared to Danish patients, English patients were, on average, 2 years older, more likely to be female, had a greater number of previous medical problems, were more likely to receive a RTSR and less likely to receive a HA, and were less likely to undergo surgery for acute trauma but more likely to have surgery for OA (Table 1).

**Table 1:** Patient characteristics for English and Danish patient cohorts. See Additional file 1 for ICD-10 codes used to identify the comorbidities listed

	**England** (*n* = 41,471)		**Denmark** (*n* = 9,268)	
**Characteristic**	**Mean/Count**	**SD/proportion**	**Mean/count**	**SD/proportion**
Age	72.6	9.9	70.6	10.1
Sex				
Male	12,177	29.4	3,037	32.8
Female	29,294	70.6	6,231	67.2
ASA grade				
1	2,659	6.4	565	6.1
2	25,883	62.4	3,195	34.5
3	12,543	30.3	1,371	14.8
4/5	386	0.9	41	0.4
Missing	0	0.0	4,096	44.2
Previous surgery				
No	34,534	83.3	7,687	82.9
Yes	6,937	16.7	1,485	16.0
Charlson comorbidity index				
0	20,289	48.9	6,008	64.8
1	10,868	26.2	842	9.1
2 +	10,314	24.9	2,418	26.1
Surgical indication				
Avascular necrosis	937	2.3	308	3.3
Acute trauma	4,439	10.7	2,491	26.9
Cuff tear arthropathy	10,430	25.2	1,888	20.4
Inflammatory arthropathy	1,482	3.6	217	2.3
Osteoarthritis	20,540	49.5	3,481	37.6
Trauma sequelae	2,654	6.4	806	8.7
Other	989	2.4	77	0.8
Procedure type				
Humeral hemiarthroplasty (HA)	6,861	16.5	3,053	32.9
Reverse total shoulder replacement (RTSR)	22,124	53.4	3,580	38.6
Anatomical total shoulder replacement (TSR)	12,486	30.1	2,635	28.4
Gastrointestinal diseases				
No	25,848	62.3	7,425	80.1
Yes	15,623	37.7	1,843	19.9
Mental health diseases				
No	32,138	77.5	8,663	93.5
Yes	9,333	22.5	605	6.5
Respiratory diseases				
No	30,077	72.5	8,047	86.8
Yes	11,394	27.5	1,221	13.2
Circulatory diseases				
No	13,266	32.0	6,249	67.4
Yes	28,205	68.0	3,019	32.6
Metabolic diseases				
No	24,914	60.1	7,726	83.4
Yes	16,557	39.9	1,542	16.6
Neurological diseases				
No	35,245	85.0	8,163	88.1
Yes	6,226	15.0	1,105	11.9
Urinary tract diseases				
No	32,925	79.4	7,994	86.3
Yes	8,546	20.6	1,274	13.7
Health hazards				
No	40,533	97.7	9,211	99.4
Yes	938	2.3	57	0.6
Obesity				
No	32,513	78.4	8,916	96.2
Yes	8,958	21.6	352	3.8
Lifestyle problems				
No	40,399	97.4	9,214	99.4
Yes	1,072	2.6	54	0.6

*SD* standard deviation

The use of the three shoulder replacement procedures per grouped surgical indication differed considerably (Additional file 1). In England, 55.0% of HAs were elective procedures compared to only 20.4% in Denmark. Patients having surgery for acute trauma were more likely to receive a HA in Denmark (77.0%) compared to England (33.3%), where they usually received a RTSR (65.7%). The distribution of use of TSR was similar across surgical indications between the two countries.

### Outcomes

**Fig. 1:**
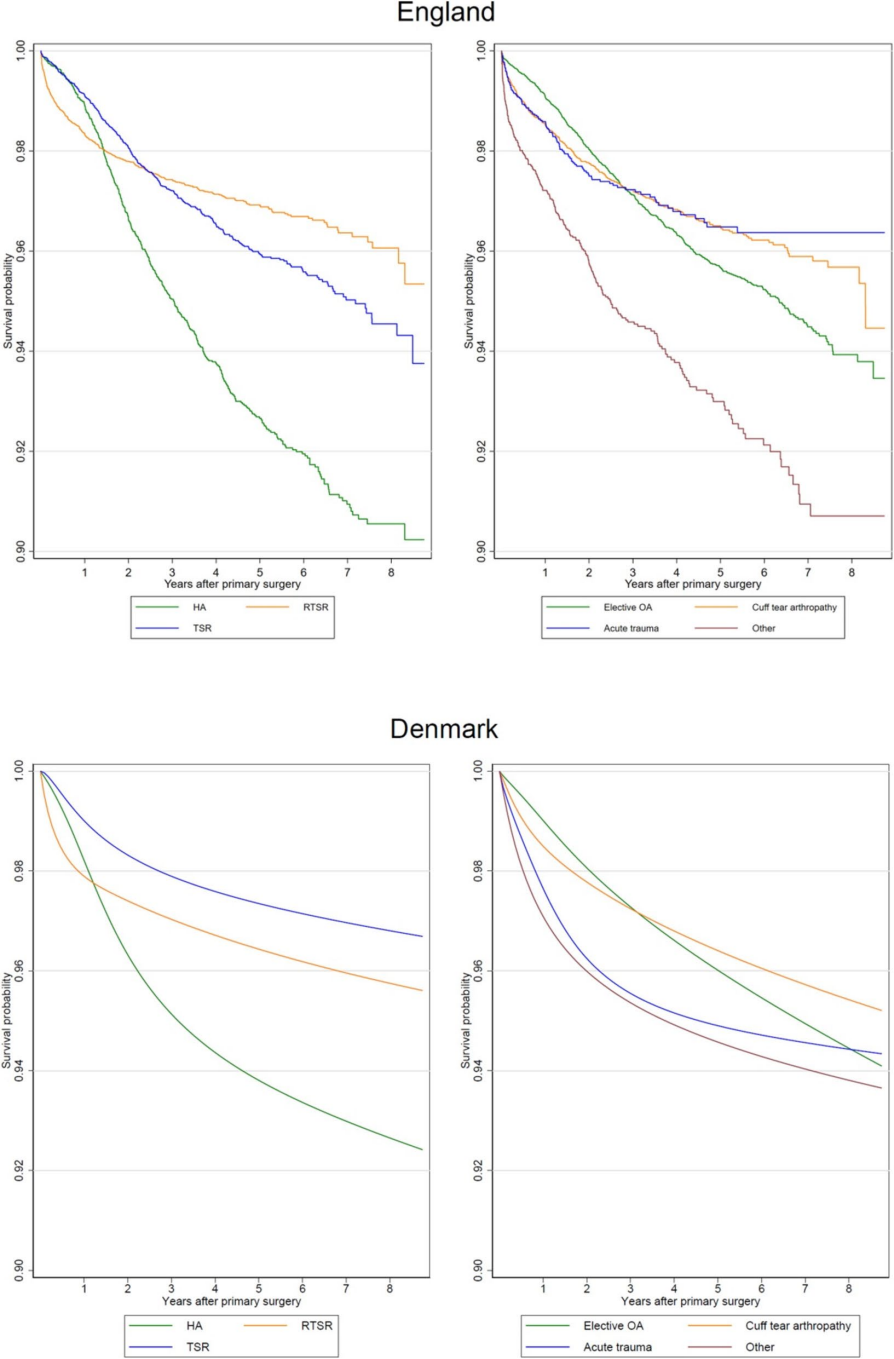
Kaplan–Meier survival curves for risk of revision by procedure type and by grouped surgical indication for each country

**Fig. 2:**
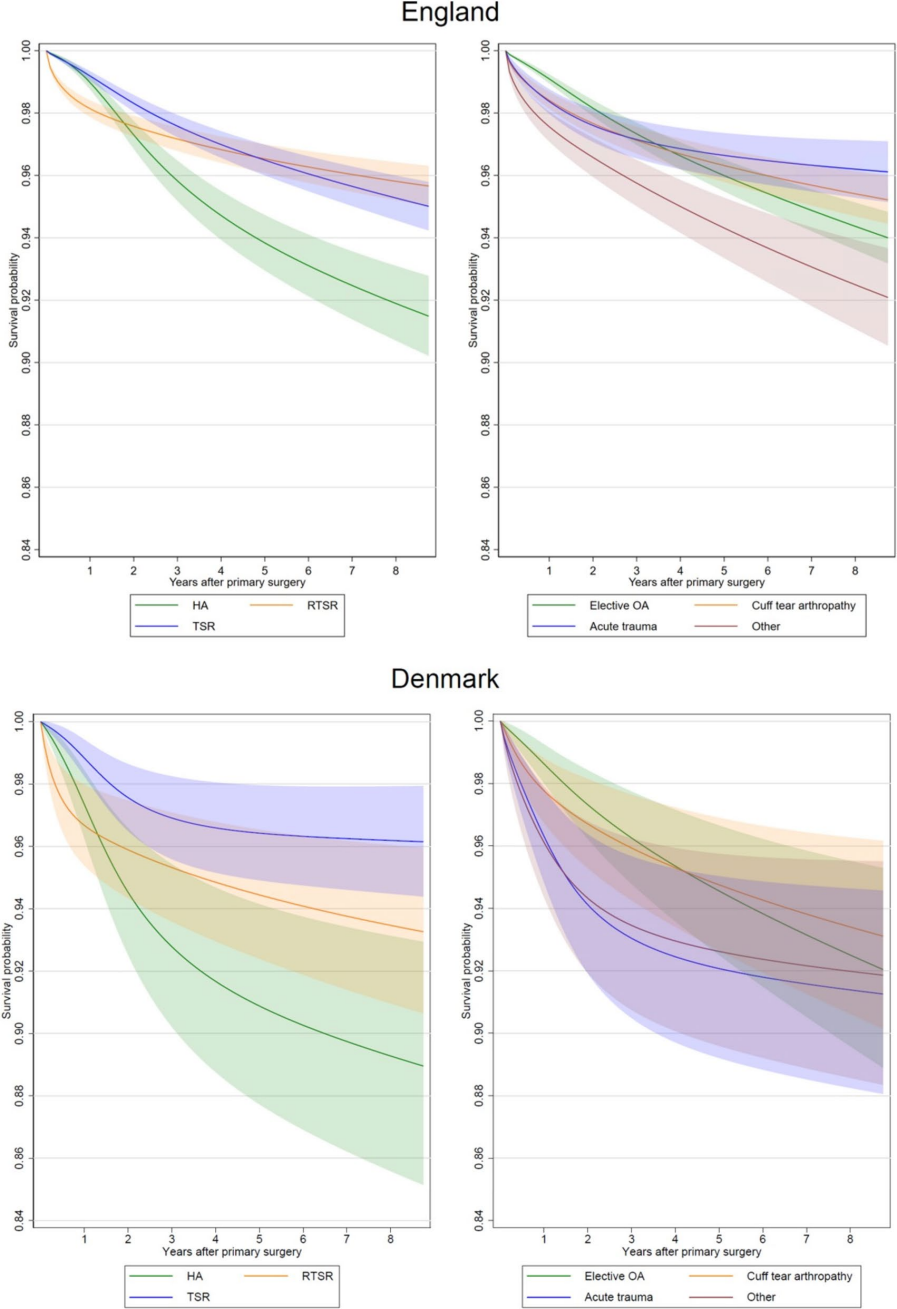
Adjusted survival curves for risk of revision by procedure type and grouped surgical indication, standardised for a 70-year-old female with a CCI of 1, for each country

In the English dataset, there were 1,429 revisions (3.5%) with a maximum follow-up of 8.75 years, a total of 154,850 years of observation time, and a revision-free survival probability of 94.3% (95% CI 93.9% to 94.7%) at 8 years. In the Danish dataset, there were 323 revisions (3.5%) with a maximum follow-up of 8.75 years, a total of 33,819 years of observation time, and a revision-free survival probability of 94.9% (95% CI 94.2% to 95.5%) at 8 years. Compared to Denmark, the unadjusted probability of revision was slightly lower for RTSR and higher for TSR and HA in England (Fig. 1). The probability of revision was considerably lower for the ‘Acute Trauma’ group in England. These patterns were maintained after confounder adjustment though there was some uncertainty with the confidence intervals being particularly wide for Denmark due to the lower sample size (Fig. 2).

**Fig. 3:**
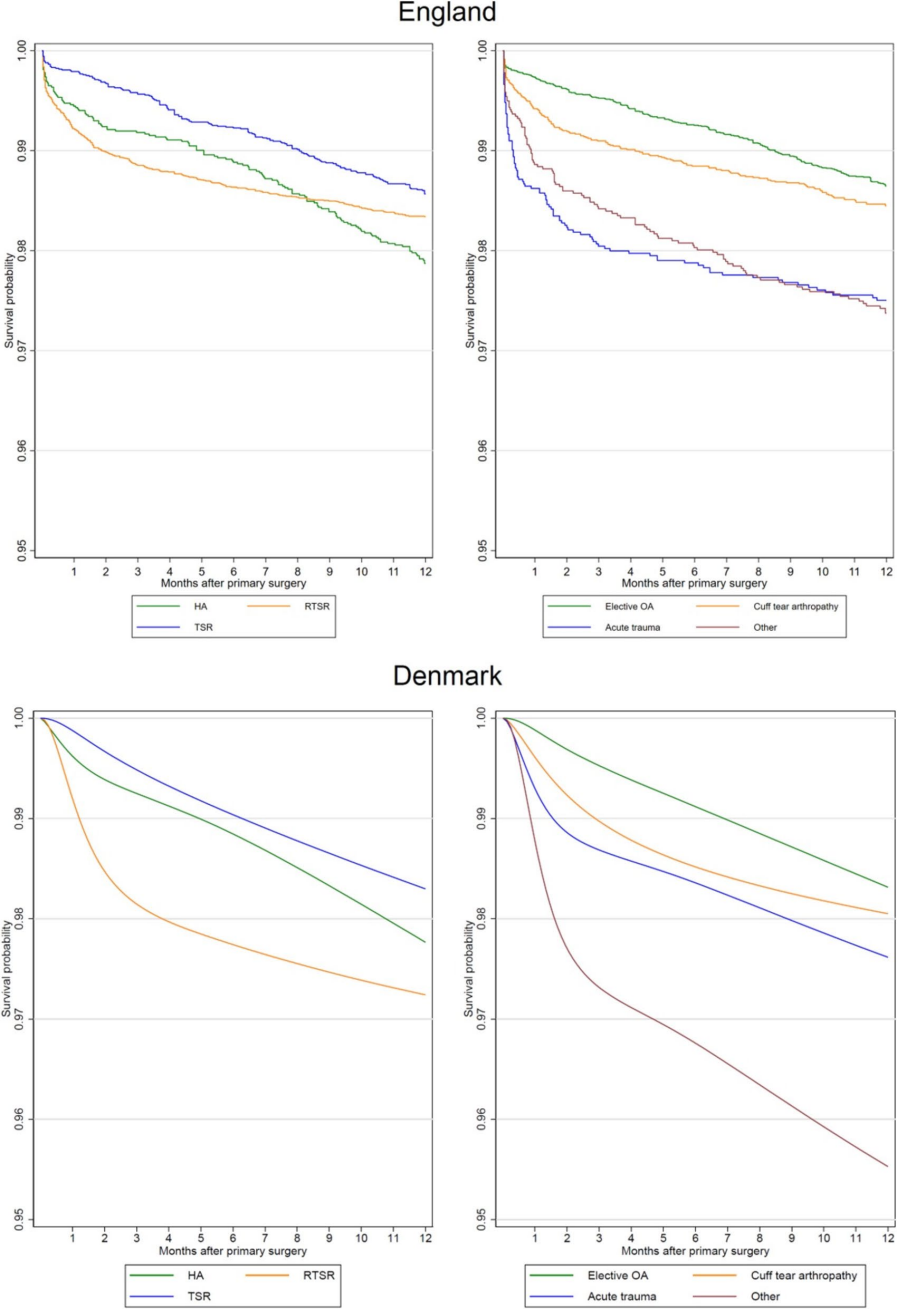
Kaplan–Meier survival curves for risk of reoperation within 1 year of surgery by procedure type and grouped surgical indication for each country

In the English dataset, there were 676 reoperations (1.6%) within a year of surgery (reoperation-free survival probability of 98.3% [95% CI 98.2% to 98.5%] at 1 year) and a total of 39,383 years of observation time. In the Danish dataset, there were 200 reoperations (2.2%) within a year of surgery (reoperation-free survival probability of 97.7% [95% CI 97.4% to 98.0%] at 1 year) and a total of 8,412 years of observation time. Compared to Denmark, the unadjusted probability of reoperation was similar for TSR and HA but lower for RTSR (Fig. 3). The probability of reoperation was similar for all surgical indications, but lower for the ‘Other’ group.

In the English dataset, there were 2,270 SAEs (5.6%) out of 40,631 procedures, whereas in the Danish dataset, there were 406 SAEs (4.5%) out of 8,948 procedures (Table 2). Overall, the unadjusted SAE rates in England were higher than in Denmark, although they were lower for HA. They were also higher for all grouped surgical indications. These differences remained for adjusted SAE point estimates, although differences were less marked, apart from those for HA which were increased further. Some uncertainty remained, as reflected in the confidence intervals.

**Table 2:** Ninety-day SAEs for the English and Danish datasets. Counts and proportions shown for unadjusted rates, and estimates with 95% confidence intervals (CI) shown for adjusted risk for a 70-year-old female with a CCI of 1, per procedure type and per grouped surgical indication

	**England**							**Denmark**						
	**Unadjusted**				**Adjusted**			**Unadjusted**				**Adjusted**		
	**No event**		**Event**		**Event**			**No event**		**Event**		**Event**		
	**Count**	**Proportion (%)**	**Count**	**Proportion (%)**	**Estimate**	**Lower CI**	**Upper CI**	**Count**	**Proportion (%)**	**Count**	**Proportion (%)**	**Estimate**	**Lower CI**	**Upper CI**
Procedure type														
Humeral hemiarthroplasty (HA)	6,473	95.2	330	4.9	4.18	3.61	4.76	2,822	93.5	195	6.5	7.01	4.84	9.19
Reverse total shoulder replacement (RTSR)	20,030	92.9	1,529	7.1	5.00	4.51	5.5	3,274	95.5	153	4.5	3.98	2.69	5.26
Anatomical total shoulder replacement (TSR)	11,858	96.7	411	3.4	3.08	2.7	3.47	2,446	97.7	58	2.3	3.11	1.94	4.27
Grouped indication														
Elective OA	19,351	96.1	795	4	3.29	2.94	3.65	3,247	97.3	89	2.7	3.13	2.03	4.22
Cuff tear arthropathy	10,982	93.9	709	6.1	4.25	3.77	4.72	1,953	96.5	71	3.5	3.03	1.91	4.16
Acute trauma	3,866	89.5	453	10.5	8.28	7.25	9.31	2,237	91.9	196	8.1	7.96	5.51	10.42
Other	4,162	93	313	7	6.05	5.22	6.88	1,105	95.7	50	4.3	5.15	3.17	7.13
Total	38,361	94.4	2,270	5.6				8,542	95.5	406	4.5			

## Discussion

### Main findings

This linked national registry and hospital study found considerable differences in case-mix and patient outcomes following shoulder replacement surgery between England and Denmark. Patients treated in Denmark were younger with fewer comorbidities and subsequently had a lower overall risk of 90-day SAEs. While reoperation rates were similar, a lower overall risk of revision surgery was present in Denmark especially for TSR and HA. However, patients having a joint replacement for acute shoulder trauma had a higher risk of revision in Denmark. All trends remained the same after adjusting for age, sex and comorbidities.

The revision surgery rates in both countries were relatively low and matched those published in other shoulder registries, representing the improved contemporary surgical techniques and implants used in shoulder replacement surgery [20, 21]. The differences in revision surgery might be explained by the different procedures chosen for each surgical indication for joint replacement. In Denmark, patients with acute trauma are more than twice as likely as they would be in England to receive a HA. In England, the majority of patients with acute trauma are offered a RTSR. The lower revision rates therefore seen in England for acute trauma might be because RTSR is a better choice, giving better patient satisfaction, or it might be that revision RTSR is harder to conduct than revision HA, giving a false sense of success. This highlights the caution needed when using revision surgery alone as an end point. While this is the accepted end point for monitoring surgeon, unit and implant performance, it does not consider patients with failing implants that might be on a long surgical waiting list, those that decide to live with a poorer outcome, or those who cannot access healthcare services for revision surgery. While both countries have a publicly funded healthcare system, revision surgery in Denmark is undertaken in a very small number of regional centres.

When compared to TSR and RTSR, HA was associated with a slightly lower adjusted risk of SAE in English patients but a considerably higher risk in Danish patients. This may also be attributed to the proportional differences observed in the use of HA in trauma and elective settings between countries.

### Comparison with other studies

There are no other published studies yet that directly compare shoulder replacement outcomes between two countries using linked national joint registry and hospital data. One study investigated international trends in shoulder replacement surgery and changes in practice using publicly available data from nine joint registries, revealing wide variation in the incidence of shoulder replacement and in procedure selection by surgical indication [1]. This study was recently repeated using publicly available data from 11 joint registries showing there is now a more uniform use of implants, although ongoing differences in shoulder replacement incidence rates remain [22].

### Strengths and limitations

A key strength of this study was the use of linked registry and hospital data that provide comprehensive coverage of public healthcare systems from both countries and enable the reporting of multiple outcomes important to patients and healthcare planners. Despite between-country differences in the capturing and coding of healthcare data (e.g. different coding systems for reoperations), a priori agreement of the definition of key variables and outcomes improved the consistency and comparability of the results. The use of regression models for case-mix adjustment allowed for a more informative between-country comparison of patient outcomes. However, the models are likely a simplification of reality, with procedure type and grouped surgical indication being accounted for in separate models, and their interactions not considered in the interest of simplicity. Moreover, we limited confounder adjustment to certain key variables, so there is likely some residual confounding, and causality cannot be inferred. As separate models were used for each country (given the inability to link the two countries’ datasets), case-mix adjustment will have been different. Finally, despite the same coding system being used for SAEs, Danish hospital data from 2019 onwards does not differentiate between inpatient and outpatient activity, meaning that SAEs may have been overestimated for this time period.

## Conclusions

Using linked national registry and hospital data from England and Denmark has identified considerable differences in patient outcomes after shoulder replacement surgery. While variation in patient demographics existed, a clinically important difference in revision rates was observed despite confounder adjustment. The likely explanation for the observed differences is variation in decision-making around the use of different shoulder replacement procedures for different surgical indications. The reasons for these decision differences are not clear but probably relate to established practices and a paucity of high-quality outcome evidence.

## Supplementary Information

Additional file 1.

Additional file 2. (RECORD checklist).

## Data Availability

Access to the data analysed in this study requires permission from the National Joint Registry Research Sub-committee. https://www.njrcentre.org.uk/research/research-requests/ contains information on research data access request to the National Joint Registry. Access to the Danish data requires permission from Statistics Denmark and the DSR. https://www.dst.dk/en & https://www.rkkp.dk/kvalitetsdatabaser/dansk-skulderalloplastik contain information on research data access requests to Statistics Denmark and the DSR. Access to the Danish data requires permission from Statistics Denmark and the DSR. https://www.dst.dk/en & https://www.rkkp.dk/kvalitetsdatabaser/dansk-skulderalloplastik contain information on research data access requests to Statistics Denmark and the DSR.

## Abbreviations

AIC: Akaike information criterion
ASA: American Society of Anaesthesiologists
BIC: Bayesian information criterion
CCI: Charlson Comorbidity Index
CRS: Civil Registration System
DNPR: Danish National Patient Registry
DSR: Danish Shoulder Arthroplasty Registry
HA: Humeral hemiarthroplasty
HES: Hospital Episode Statistics
ICD-10: International Classification of Diseases, 10th revision
NHS: National Health Service
NJR: National Joint Registry
NOMESCO: Nordic Medico-Statistical Committee
OA: Osteoarthritis
OPCS-4: Office of Population Censuses and Surveys classification of surgical operations and procedures, fourth revision
RTSR: Reverse total shoulder replacement
SAE: Serious adverse event
TSR: Anatomical total shoulder replacement
