# Supplementary material for: A comparative study of shoulder replacement outcomes using linked national registry and hospital data from England and Denmark
Source: BMC Med. 2025 Mar 26;23:180. doi: 10.1186/s12916-025-04003-3 (PMC11948730; doi:10.1186/s12916-025-04003-3)
Supplement: Supplementary file 1 — Additional file 1. [file 12916_2025_4003_MOESM1_ESM.docx]

**Additional file 1**

## Data flowcharts


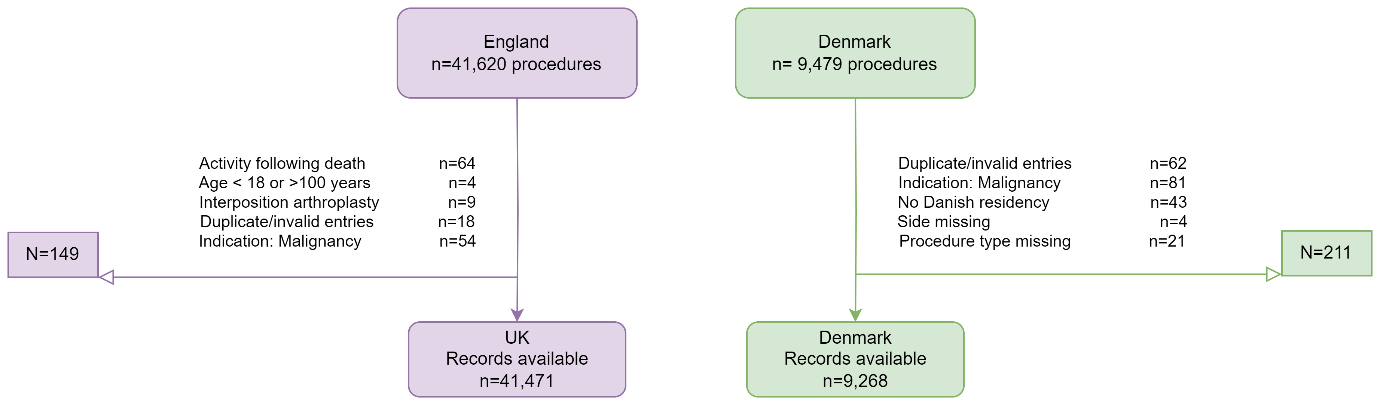


## ICD-10 codes for comorbidities

| **Comorbidity** | **ICD-10 codes** |
| --- | --- |
| Gastrointestinal diseases | K00-K93 |
| Mental health diseases | F00-F99 |
| Respiratory diseases | J00-J99 |
| Circulatory diseases | I00-I99 |
| Metabolic diseases | E00-E90 (not E66) |
| Neurological diseases | G00-G99 |
| Urinary tract diseases | N00-N99 |
| Health hazards | Z55-Z65 |
| Obesity | E66 |
| Lifestyle problems | Z72 |

## ICD-10 codes for serious adverse events

| **Event** | **ICD-10 codes** |
| --- | --- |
| Pulmonary embolism | I26 |
| Myocardial infarction | I21,I22 |
| Cerebrovascular event | I60,I61,I62,I63,I64 |
| Acute kidney injury | N17 |
| Lower respiratory tract infection | J12,J13,J14,J15,J16,J18,J22,J86,J440,J851,J690 |
| Urinary tract infection | N10,N300,N308,N309,N390 |
| Death | *Mortality data* |

## OPCS-4 and NOMESCO codes for reoperations

| **Procedure type** | **OPCS-4 codes** | **Anatomy codes** | **NOMESCO codes** |
| --- | --- | --- | --- |
| SAD/ACJ excision | O291 |  | NBG09  NBG19 |
|  | W572,W844 | Z812 | NBH5* |
|  | T621,T622,T626,T628,T629 | Z814,Z891 |  |
| Rotator cuff repair | T791,T793,T794,T795 |  | NBL49 |
|  | T641,T642,T643,T744,T648,T649,T67,T68 | Z742 |  |
| MUA +-release | W911,W913,W918,W919,W781,W784, Y421, Y428, Y429 | Z813, Z814, Z891 | NBH3*  NBT19 |
| Washout/debridement | W80, W811, W812, W813, W815, W713, Y223, Y311, Y318, Y319, Y321 | Z813, Z814, Z891 | NBW*, NBS* |
|  | W18 | Z691, Z692, Z693, Z694 |  |
| Synovectomy | W691, W692, W693, T711 | Z813, Z814, Z891 | NBF* |
| Osteomyelitis surgery | W18 | Z691, Z692, Z693,Z694 | NBS29 |
| Complex reconstruction | O108, O109 |  | NBN* |
| Bone resection | W068, W069, W091, W092, W093, W094, W095, W096, W097, W098, W099 | Z691, Z692, Z693 | NBK* |
| Arthroscopy or other soft tissue | Y528, Y767, W816, W817, W818, W819, W83, W843, W845, W846, W847, W848, W868, W869, W881, W888, W889, W891, W898, W899, O198, O199, W714, W718, W719, W694, W695, W698, W699, W711, W712, T645, T651, T658, T659, T701, T702, W562, W563, W564, W568, W569 | Z812, Z813, Z814, Z891 | NBA*, NBM*, NBH4*,  NBH9*, NBL* |
| Surgery for instability | O27 |  | NBE*,  NBH7* |
|  | W77 (Not W776), W841, W842 |  |  |
|  | W72, W73, W74, W75 | Z813, Z814, Z891 |  |
| Reduction of dislocation | W652, W658, W689, W662, W668, W669, W672, W674, W678, W679 | Z813, Z814, Z891 | NBH0*, NBH2* |
| Fixation of periprosthetic fracture | W19 (not W191), W20, W21, W22, W23, W24 (not W241), W25, W26, W651, W653, W654, W656, W661, W663, W664, W671, W673, W677, W332, O172, O173, O175, O178, O179 | Z691, Z692, Z693, Z813, Z814, Z891 | NBJ* |

## Procedure type by grouped indication

|  | **Grouped surgical indication** | | | | | | | | | |
| --- | --- | --- | --- | --- | --- | --- | --- | --- | --- | --- |
|  | **England** | | | | | **England** | | | | |
| **Procedure type** | Elective | Cuff tear arthropathy | Acute trauma | Other | Total | Elective | Cuff tear arthropathy | Acute trauma | Other | Total |
| HA | 3771 | 643 | 1476 | 971 | 6861 | 622 | 175 | 1919 | 337 | 3053 |
|  | *55.0* | *9.4* | *21.5* | *14.2* |  | *20.4* | *5.7* | *62.9* | *11.0* |  |
|  | *18.4* | *5.4* | *33.3* | *21.2* |  | *17.9* | *8.3* | *77.0* | *28.3* |  |
| RTSR | 5618 | 10667 | 2917 | 2922 | 22124 | 594 | 1855 | 548 | 583 | 3580 |
|  | *25.4* | *48.2* | *13.2* | *13.2* |  | *16.6* | *51.8* | *15.3* | *16.3* |  |
|  | *27.4* | *89.5* | *65.7* | *63.8* |  | *17.1* | *88.1* | *22.0* | *49.0* |  |
| TSR | 11151 | 602 | 46 | 687 | 12486 | 2265 | 75 | 24 | 271 | 2635 |
|  | *89.3* | *4.8* | *0.4* | *5.5* |  | *86.0* | *2.8* | *0.9* | *10.3* |  |
|  | *54.3* | *5.1* | *1.0* | *15.0* |  | *65.1* | *3.6* | *1.0* | *22.8* |  |
| Total | 20540 | 11912 | 4439 | 4580 | 41471 | 3481 | 2105 | 2491 | 1191 | 9268 |

First row has frequencies. Second row has *row percentages*, third row has *column percentages*.

## Overall revision probabilities

Grouped (1-Kaplan Meier) estimate of failure probability for revision for each country.

| Time (years) | Failure (%) | | | | | |
| --- | --- | --- | --- | --- | --- | --- |
|  | England | | | Denmark | | |
|  | Estimate | Lower CI | Upper CI | Estimate | Lower CI | Upper CI |
| 1 | 1.3 | 1.2 | 1.5 | 1.6 | 1.4 | 1.9 |
| 2 | 2.4 | 2.2 | 2.5 | 2.8 | 2.5 | 3.2 |
| 3 | 3.1 | 2.9 | 3.3 | 3.3 | 3.0 | 3.8 |
| 4 | 3.8 | 3.6 | 4.0 | 4.1 | 3.6 | 4.6 |
| 5 | 4.3 | 4.1 | 4.6 | 4.5 | 4.0 | 5.0 |
| 6 | 4.7 | 4.5 | 5.0 | 4.9 | 4.4 | 5.5 |
| 7 | 5.3 | 5.0 | 5.7 | 5.0 | 4.4 | 5.6 |
| 8 | 5.7 | 5.4 | 6.1 | 5.1 | 4.5 | 5.8 |

## Overall reoperations probabilities

Grouped (1-Kaplan Meier) estimate of failure probability for reoperations for each country.

| Time (months) | Failure (%) | | | | | |
| --- | --- | --- | --- | --- | --- | --- |
|  | England | | | Denmark | | |
|  | Estimate | Lower CI | Upper CI | Estimate | Lower CI | Upper CI |
| 1 | 0.6 | 0.5 | 0.7 | 0.5 | 0.4 | 0.7 |
| 2 | 0.8 | 0.7 | 0.9 | 0.9 | 0.7 | 1.1 |
| 3 | 0.9 | 0.8 | 1.0 | 1.1 | 0.9 | 1.3 |
| 4 | 1.0 | 0.9 | 1.1 | 1.3 | 1.1 | 1.5 |
| 5 | 1.1 | 1.0 | 1.2 | 1.4 | 1.1 | 1.6 |
| 6 | 1.2 | 1.1 | 1.3 | 1.6 | 1.3 | 1.8 |
| 7 | 1.2 | 1.1 | 1.3 | 1.7 | 1.5 | 2.0 |
| 8 | 1.3 | 1.2 | 1.4 | 1.8 | 1.5 | 2.1 |
| 9 | 1.4 | 1.3 | 1.5 | 1.9 | 1.6 | 2.2 |
| 10 | 1.5 | 1.4 | 1.6 | 2.0 | 1.8 | 2.4 |
| 11 | 1.6 | 1.5 | 1.7 | 2.1 | 1.9 | 2.5 |
| 12 | 1.7 | 1.6 | 1.8 | 2.3 | 2.0 | 2.6 |
